# A Laparoendoscopic Single-Site Surgery Approach to Mesh Sacrohysteropexy

**DOI:** 10.1155/2013/641675

**Published:** 2013-02-25

**Authors:** Fariba Behnia-Willison, Anirudha Garg, Marc J. N. C. Keirse

**Affiliations:** Department of Obstetrics, Gynaecology and Reproductive Medicine, Flinders University and Flinders Medical Centre, Bedford Park, SA 5042, Australia

## Abstract

Although laparoendoscopic single-site surgery (LESS) has spread across surgical disciplines, this has not been the case for the repair of uterovaginal prolapse. We describe the use of this technique for mesh sacrohysteropexy to correct a global prolapse classified as stage II on the pelvic organ prolapse quantification (POP-Q) system. The procedure involved intraoperative modification of a commercially available single incision port. At the 18 months followup, the patient was free of symptoms and had no objective prolapse.

## 1. Introduction

Laparoendoscopic single-site surgery (LESS), also variously known as single incision surgery or single-port surgery, is a minimally invasive procedure which is based on the premise that the use of a single umbilical incision results in less postoperative pain and better cosmetic results than the traditional, multiport laparoscopic surgery. Single incision laparoendoscopic surgery is increasingly being used for a variety of surgical procedures. Although its use for tubal ligation dates back to more than 40 years [1], compared with other surgical specialties, there is still relatively little literature on outcomes of single-port surgery in gynecological surgery. A recent literature review indicated that only 4 percent of all laparoendoscopic single-site procedures had been performed by gynecologists [2]. Most gynecological procedures performed through single incision, apart from tubal ligation, have been hysterectomies, oophorectomies, and ovarian cystectomies [3]. Here we report on the use of the single-port technique for mesh sacrohysteropexy. To the best of our knowledge, mesh sacrohysteropexy through a single-port approach has not been reported previously.

## 2. Patient Presentation

The patient was a 70-year-old woman with a body mass index of 26, who was para 3 having had 3 vaginal births. She presented with symptomatic global prolapse and had found no relief from conservative management including pelvic floor exercises and vaginal estrogens. At her request, she was scheduled for a laparoendoscopic single-site mesh sacrohysteropexy followed by anterior and posterior vaginal repair with biological mesh augmentation. Her pelvic organ prolapse quantification (POP-Q) [4], as determined intraoperatively, was GH 4 cm, C +1.5, Aa 0, Ba +0.5, Ap 0, Bp −3, D 8, TVL 10, and PB 1.5 cm resulting in a global prolapse stage score of II. 

## 3. Intervention

**Figure 1:**
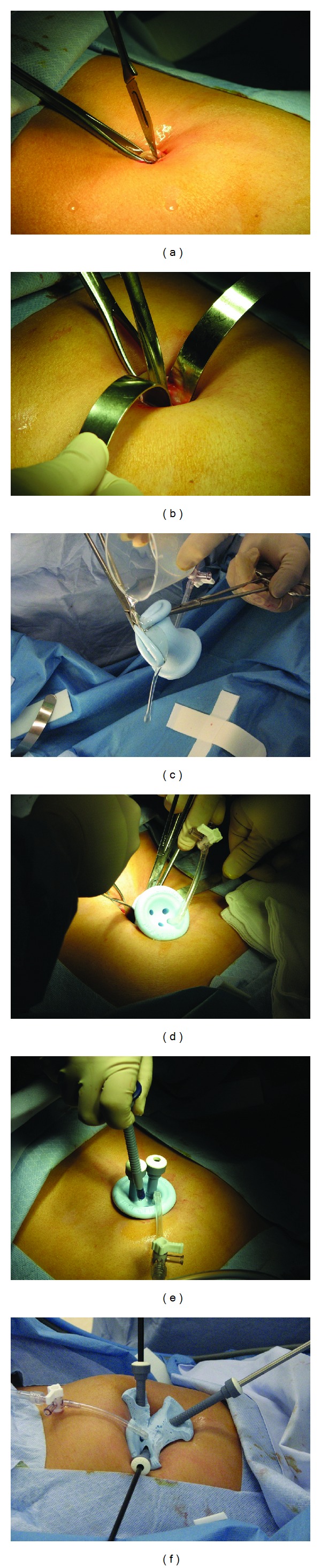
Insertion and modification of the Covidien SILS port: (a) incision to gain access to the peritoneal cavity via the umbilicus; (b) using S retractors to establish pneumoperitoneum; (c) lubricating the SILS port with paraffin wax; (d) grasping the SILS port using two Blake forceps and insertion into the umbilicus with the forceps as a guide; (e) insertion of the trocars and insufflation of the abdomen; and (f) Y-shaped modification of the port by 3 cuts made at an angle of 120 degrees from each other to allow for greater maneuverability.

The procedure was performed under general anesthesia in lithotomy position. After skin preparation and draping, a periumbilical infiltration was made with 5 mL injections of 0.5% bupivacaine and adrenalin at each of the 3, 6, 9, and 12 o' clock positions. As shown in Figure 1, a 15–20 mm vertical trans-umbilical skin incision was made. The rectus sheath was lifted and held with two Littlewood's graspers, and the fascia was incised with a scalpel. The incision in the fascia was extended bluntly using artery forceps before inserting two S retractors. A Covidien SILS port (Tyco Healthcare, Lane Cove, NSW, Australia) was inserted through the incision by grasping the base of the port with two Blake forceps. The Blake forceps were arranged so that the first forceps was positioned from the midsection of the port to the leading edge and the second from the trailing edge to the mid-section with the tip of the second clamp meeting the heel of the first. The port was lubricated with paraffin, and the first clamp was inserted through the incision directed towards the right lateral abdominal wall. When the heel of the first clamp had entered through the sheath, it was removed while continuous pressure was exerted on the second clamp in an arc-like motion until the lower edge of the port had entered completely through the sheath. The port was then modified by making a Y-shaped cut with a scalpel between the three channels, at 120 degrees from each other, to enable greater movement of the instruments (Figure 1). This modification not only increased the range of motion of instruments, but it also improved ergonomics and reduced clash of the instruments. The trocars were then inserted.

Once pneumoperitoneum was established, the operation commenced with the insertion of a bariatric 5 mm, 30-degree laparoscope, and two instruments. The Covidien SILS port provides three 5 mm channels, one of which can be upsized to 12 mm. There is also a conduit for CO_2_ insufflation (BOC, SA, Australia). The single-port procedure was performed similarly to conventional laparoscopic surgery. However, because of ergonomics and instrument design, crossing of instruments was possible and at times necessary.

The patient was tilted to the left. The sigmoid colon was gently pushed to the left with a blunt bowel grasper and the right ureter, and iliac vessels were identified. The peritoneum over the sacral promontory was then grasped and lifted, and an incision was made from the top of the sacral promontory to the back of the cervix at the most caudal level of the insertion of the uterosacral ligaments. 

Next, a polypropylene type 1 monofilament macroporous nonabsorbable mesh (AMS, USA) was used to suspend the cervix from the sacral promontory. The length of mesh was measured and tailored to the patient (15 cm). The mesh was anchored to the posterior cervix with five absorbable ProTack fasteners (AbsorbaTack Fixation Device, Covidien). The other end of the mesh was then fixed to the sacral promontory utilising 5 mm non-absorbable helical fasteners (ProTack Fixation Device, Covidien) to elevate the uterus. The aim is to lift the cervix at least 6–8 cm above the level of the introitus to allow shortening of the mesh and subsequent fibrosis. The entire length of the mesh was covered with peritoneum closed with Vicryl 2-0 using a Covidien Endostitch device.

Hemostasis was achieved, and the diameter of the ureter was inspected to exclude obstruction-induced dilatation. As is common practice with laparoscopic pelvic floor repairs in our department, a cystoscopy was performed after the procedure to observe ureteric patency before the trocars were removed. The sheath within the trans-umbilical port site was identified and sutured using long-absorbable sutures (PDS) before skin closure. The overall operating time was 70 minutes.

After completion of the hysteropexy, an anterior and posterior vaginal repair was undertaken with biological mesh. A vaginal pack and indwelling catheter were inserted, and the patient was transferred to recovery with calf compressors. Low molecular weight heparin (Enoxaparin) was commenced 8 hours after the operation. There were no intraoperative or postoperative complications. No analgesia was required postoperatively, and the patient was discharged after defecation 2 days after the intervention. 

## 4. Followup

One week post-operatively, the patient had a port site infection, which was treated and resolved with oral antibiotics. She had no prolapse at the 6-week followup, and the size of the umbilical scar was 1.5 cm. At 6-month followup, she remained free of symptoms. The port site had healed to 0.5 cm. Objective evaluation showed GH 2 cm, C −8, Aa −3, Ap −3, Ba −3, Bp −3, TVL 10, PB 3 cm, indicating the absence of any prolapse. At 6 months, the patient still rated her satisfaction as 9 on a 10-point visual analogue scale, mainly, because of the absence of post-operative pain, quick recovery, early return to day-to-day life, and cosmesis.

On followup at 12 months and 18 months there were no symptoms and no objective prolapse on POPQ assessment. There was no evidence of mesh erosion or any other complication. The umbilical scar was no longer visible. The patient used vaginal estrogen cream twice weekly and had regular pelvic floor exercises. 

## 5. Discussion

Studies of nongynecological patients show a comparable rate of minor complications, such as wound infection, with laparoendoscopic single-site surgery as those found with standard laparoscopy [5, 6]. Van den Boezem and Seitses reported 4 cases of wound infection in 50 laparoendoscopic single-site colorectal operations [7]. We observed a similar number of infections: 6 of 100 patients undergoing gynecological laparoendoscopic single-site surgery developed port-site infections [8]. However, this is a minor complication that is easily treated with oral antibiotics. It has been reported that laparoendoscopic single-site procedures require a longer operating time than conventional laparoscopic procedures. This would seem to apply to laparoendoscopic single-site sacrohysteropexy too. In our experience, the time to perform a conventional laparoscopic sacrohysteropexy is 20 to 40 minutes [8], compared with 70 minutes for the laparoendoscopic single-site sacrohysteropexy. However, operating times decrease with increasing experience of the surgeon [8]. So, we postulate that the time for laparoendoscopic single-site sacrohysteropexy will also decrease with experience. 

As any procedure, laparoendoscopic single-site surgery has some limitations, such as the necessary surgical skill and the need for careful selection of patients. A relatively low body mass index is required, and there should be no adhesions from previous abdominal surgery. Furthermore, a laparoendoscopic single-site pelvic floor repair is a physically taxing procedure; there can be a crowding of instruments; the relative novelty of the laparoendoscopic single-site instruments means that there is still room for improvement to maximize ergonomics. 

## 6. Conclusion

This paper shows that laparoendoscopic single-site surgery is a feasible substitute for conventional laparoscopic sacrohysteropexy. The better cosmetic result and shorter recovery time resulted in high patient satisfaction. As interest in laparoendoscopic single-site surgery grows, the range of surgical procedures that use this approach is also likely to increase. Currently, most are still confined to specialized centers as the jury is still out on their value and their relative merit compared with conventional laparoscopic surgery in routine clinical practice. Nonetheless, this paper shows the feasibility of this technique to resolve complex gynecological problems, such as uterus-sparing prolapse repairs.
